# Unfavorable and favorable changes in modifiable risk factors and incidence of coronary heart disease: The Whitehall II cohort study

**DOI:** 10.1016/j.ijcard.2018.07.005

**Published:** 2018-10-15

**Authors:** Marianna Virtanen, Jussi Vahtera, Archana Singh-Manoux, Marko Elovainio, Jane E. Ferrie, Mika Kivimäki

**Affiliations:** a Department of Public Health and Caring Sciences, Uppsala University, Sweden; b Department of Public Health, University of Turku, Turku University Hospital, Turku, Finland; c French National Institute for Health & Medical Research, Inserm, U1018, Villejuif, France; d Department of Psychology and Logopedics, University of Helsinki, Helsinki, Finland; e Department of Epidemiology and Public Health, University College London, London, UK

**Keywords:** Ischemic heart disease, Cholesterol, Hypertension, Behavioral, Psychosocial, Pseudo-trial

## Abstract

**Background:**

Few studies have examined long-term associations of unfavorable and favorable changes in vascular risk factors with incident coronary heart disease (CHD). We examined this issue in a middle-aged disease-free population.

**Methods:**

We used repeat data from the Whitehall II cohort study. Five biomedical, behavioral and psychosocial examinations of 8335 participants without CHD produced up to 20,357 person-observations to mimic a non-randomized pseudo-trial. After measurement of potential change in 6 risk factors twice (total cholesterol, blood pressure, smoking, overweight, psychological distress, problems in social relationships), a 5-year follow-up of CHD was undertaken.

**Results:**

Incidence of CHD was 7.4/1000 person-years. Increases from normal to high cholesterol (hazard ratio, HR = 1.59, 95% CI 1.26–2.00) and from normal to high blood pressure (HR = 1.64, 95% CI 1.33–2.03), as compared to remaining at the normal level, were associated with increased risk of CHD. In contrast, decreases from high to low levels of cholesterol (HR = 0.73, 95% CI 0.58–0.91), psychological distress (HR = 0.68, 95% CI 0.51–0.90), and problems in social relationships (HR = 0.65, 95% CI 0.50–0.85), and quitting smoking (HR = 0.49, 95% CI 0.29–0.82) were associated with a reduced CHD risk compared to remaining at high risk factor levels. The highest absolute risk was associated with persistent exposure to both high cholesterol and hypertension (incidence 18.1/1000 person-years) and smoking and overweight (incidence 17.7/1000 person-years).

**Conclusions:**

While persistent exposures and changes in biological and behavioral risk factors relate to the greatest increases and reductions in 5-year risk of CHD, also favorable changes in psychosocial risk factors appear to reduce CHD risk.

## Highlights

• We examined the association between changes in biological, behavioural and psychosocial factors and the incidence of CHD.
• Increases in cholesterol and blood pressure, as compared to remaining at the normal level were associated with risk of CHD.
• Favorable changes in cholesterol, distress and social relationships, as well as quitting smoking decreased the CHD risk.
• The absolute risk was highest in a combination of persistent high cholesterol and hypertension, or smoking and overweight.
• In addition to biological and behavioral factors, favorable changes in psychosocial risk factors appear to reduce CHD risk.

## 1 Introduction

In 2016, cardiovascular diseases were the leading cause of years of life lost globally [1] and the leading risk factors to years lost due to death or disability were smoking, high body mass index and high systolic blood pressure [2]. Even 80% of premature heart disease is claimed to be preventable via risk factor reduction [3]. Established modifiable biological and behavioral risk factors included in clinical guidelines are dyslipidemia, smoking, hypertension, and overweight [4], although recently, psychosocial factors such as depression and psychosocial stress have been introduced as potential risk factors [4]. The best evidence comes from randomized controlled trials (RCTs) of lipid-lowering and antihypertensive therapies, smoking cessation and weight loss. In contrast, the evidence on psychosocial risk factors and CHD is mostly based on observational data in which the exposure assessed only once [[4], [5], [6], [7], [8], [9]].

Due to ethical issues regarding manipulation of some risk factors, RCTs can only address the effects of risk reduction. Observational data provide an opportunity to examine the ‘natural course’ of risk factors; i.e., both their onset and reversal over time, as well as persistent exposure. Recently, studies have used repeat observational data to mimic the design of a trial and create ‘pseudo-trials’ [10,11] although we are not aware of such studies with CHD as an outcome. Repeat observational data analyzed as pseudo-trials, using clearly defined participant inclusion and exclusion criteria, can be used to address questions on both disease etiology and risk reduction. For example, persistent exposure to a certain risk factor can be compared to persistent non-exposure and similarly, onset and reversal of the risk factor can be examined.

Here, we analyzed data from five clinical study waves of the prospective observational Whitehall II cohort study as pseudo-trials assessing the association of the natural course of major biological, behavioral and psychosocial risk factors with the incidence of CHD in a disease-free population.

## 2 Materials and methods

### 2.1 Study population and design

Recruitment to the Whitehall II study took place between 1985 and 1988 among all office staff, aged 35 to 55 years, in 20 London-based Civil Service departments [12]. The response rate was 73% (6895 men and 3413 women). Since baseline, a total of five biomedical data collection waves have been completed. University College London Medical School Committee on the Ethics of Human Research approved the protocol and written informed consent was obtained from all participants. Whitehall II data, protocols, and other metadata are available to bona fide researchers for research purposes (http://www.ucl.ac.uk/whitehallII/data-sharing).

As presented in Supplemental Fig. 1, Supplemental Fig. 2, we used data from five clinical study waves (1, 3, 5, 7, 9) which were arranged into three nested cohorts (‘pseudo-trials’) according to predefined inclusion and exclusion criteria, as previously described [11]; 1-3-5, 3-5-7, and 5-7-9. We included only participants who were free of CHD at the first (Tx) and second (Tx + 1) study waves. The outcome was incident CHD between Tx + 1 and Tx + 2. This study design resulted in up to 20,357 person-observations from 8335 individuals participating in at least one cycle (1600 participated in one cycle only, 1448 participated twice and 5287 participated in all three cycles); with 799 IHD events over 107,907 person years (average rate 7.4/1000 person-years). Mean total follow-up from Tx to Tx + 2 was 11.0 (SD = 0.7) years; the assessments of exposures (Tx to Tx + 1) were approximately 5.6 (SD = 0.8) years apart; and the mean follow-up from Tx + 1 to CHD incidence by Tx + 2 was 5.3 (SD = 0.9) years.

To analyze loss to follow-up, we compared the baseline (study entry, wave 1) characteristics between each nested study cohort and all 10,187 Whitehall II study respondents without CHD at study entry (Supplemental Table 1). Apart from participants with low socioeconomic status and non-white ethnic background being somewhat under-represented among the nested study cohorts, we did not find major differences in the baseline characteristics.

### 2.2 Ascertainment of coronary heart disease

For ascertainment of CHD death, participants were flagged by the British National Health Service (NHS) Central Registry, who notified us of the date and cause of all deaths, classified as coronary: ICD-9 (*International Classification of Diseases*, 9th edition) codes 410–414 or ICD-10 (*International Classification of Diseases*, 10th edition) codes I20–I25 present on the death certificate. Nonfatal CHD included first nonfatal myocardial infarction (MI) or first definite angina. Non-fatal MI was defined following MONICA criteria [13] based on study electrocardiograms, hospital acute ECGs, and cardiac enzymes. Incident angina was defined on the basis of clinical records and nitrate medication use, excluding cases based solely on self-reported data without clinical verification. The cases were ascertained from participants' general practitioners, information extracted from hospital medical records by study nurses, or data from the NHS Hospital Episode Statistics (HES) and death register databases obtained after linking the participants' unique NHS identification numbers to this national database. All self-reported cases without clinical verification were excluded.

### 2.3 Measurement of risk factors

Classic CHD risk factors included clinical measurements of total cholesterol (>6.2 mmol/l defined as high), blood pressure (systolic ≥140 mm Hg or diastolic ≥90 mm Hg defined as hypertension), and overweight, based on body mass index (BMI) of ≥25 kg/m^2^, calculated from measurements of weight and height at the study clinic. Smoking was based on survey responses and classified as never, ex, and current smoking at each wave.

Emerging psychosocial risk factors included psychological distress and problems in social relationships. Psychological distress was measured using the General Health Questionnaire (GHQ-30) [14], with caseness (high psychological distress) defined as having a score of 5 or more. The nature of close social relationships was ascertained using a 4-item ‘negative aspects of close relationships’ scale from the Close Persons Questionnaire [15,16]. The items were, for example: How much in the last 12 months … “did this [closest] person give you worries, problems and stress?”, with response alternatives 1 = not at all; 2 = little; 3 = quite a lot; 4 = a great deal. A mean of the items was calculated and dichotomized as <2 (low level of negative aspects) and ≥2 (high level of negative aspects in close relationships). The measure was not available at wave 3. In this part of analyses, the study design included nested cohorts from waves 1-2-5, 2-5-7, and 5-7-9 with the corresponding follow-up for incident CHD.

Covariates included age, sex, socioeconomic status (three classes based on occupational position), ethnicity (white, South Asian, black, other; classified as white versus non-white), marital status (married/co-habited versus not) and self-reported long-standing illness (yes versus no) were based on survey responses at the beginning of incident CHD follow-up (Tx + 1).

### 2.4 Statistical analyses

Data from the three pseudo-trials were first pooled (see Supplemental Fig. 1, Supplemental Fig. 2), after which we examined associations between exposure to risk factors at Tx and Tx + 1 (and their changes), and the incidence of CHD between Tx + 1 and Tx + 2 in a population free of CHD at Tx + 1. We used two measurements of each risk factor to classify the participants into four groups; persistent/repeated absence of risk (normal at Tx and Tx + 1), onset of risk (normal at Tx and high at Tx + 1), reversal of risk (high at Tx and normal at Tx + 1) and persistent/repeated presence of risk factor (high at Tx and high at Tx + 1. For smoking there were five groups; persistent never-smoker, persistent ex-smoker, relapse/onset, quitter, and persistent smoker. To examine absolute levels of CHD incidence, we calculated the incidence rate per 1000 person-years. We used Cox proportional hazard models in which each participants was followed from the date of Tx + 1 to the earliest out of a CHD event, death, or end of follow-up (Tx + 2). We calculated the Hazard ratios and their 95% confidence intervals using sandwich variance estimate to control for intra-individual correlation between repeated measurements nested within participants (i.e., a study participant could contribute to more than one cycle of observations in the dataset) and to take into account non-independence of the within-participant observations when estimating standard errors.

First, we compared all other groups with participants with persistent/repeated non-exposure to a risk factor. Then we performed an additional analysis to assess the difference between those who had persistent/repeated exposure to the risk factor and those in whom the risk factor had reversed. Finally, within the biological (cholesterol and blood pressure), behavioral (smoking and overweight) and psychosocial (distress and relationship problems) risk factor groups, we compared those who were persistently or repeatedly exposed to both risk factors and those who had an onset of either one of the risk factors, to those who were repeatedly or persistently non-exposed to either of the two risk factors. The analyses were serially adjusted for: 1) age and sex; 2) additionally socioeconomic status, ethnicity, and marital status; 3) additionally self-reported long-standing illness. Sensitivity analyses included the adjusted of model 3 for the number of study cycle and analyzing the results among participants of >60 years of age. SAS version 9.4 (SAS, Cary, NC, USA) was used for all analyses.

## 3 Results

Baseline characteristics of the nested study cohorts (Table 1) show higher age, higher prevalence of hypertension and overweight and lower prevalance of smoking, psychological distress and relationship problems in later cohorts, while the prevalence of high total cholesterol followed an inversed U-shape.

**Table 1:** Baseline characteristics based on observations among each nested study cohort.

Characteristic	Cohort 1		Cohort 2		Cohort 3	
	No. of obs.^a^	Mean (S.D.) or %	No. of obs.^a^	Mean (S.D.) or %	No. of obs.^a^	Mean (S.D.) or %
Mean age^b^ (y)	8012	49.5 (6.1)	6336	55.7 (6.0)	6009	60.9 (5.9)
Age range^b^ (y)	8012	39–63	6336	44–68	6009	50–74
Sex (% men)	8012	69.0	6336	70.6	6009	70.0
Total cholesterol (mean, S.D. mmol/l)	7551	5.9 (1.1)	5424	6.4 (1.1)	5017	5.9 (1.06)
Total cholesterol (% high)	7551	34.2	5424	56.1	5017	34.2
Systolic blood pressure (mean, S.D. mm Hg)	7618	122.5 (14.2)	5487	120.0 (13.2)	5095	122.4 (16.1)
Diastolic blood pressure (mean, S.D. mm Hg)	7617	76.6 (9.9)	5486	79.4 (9.2)	5094	77.3 (10.4)
Hypertension (% yes)	7617	15.8	5486	15.0	5095	19.5
Smoking (%): never	4080	52.4	3151	52.4	2763	51.7
Ex	2615	33.6	2135	35.5	2056	38.5
Current	1096	14.1	728	12.1	525	9.8
Body mass index (mean, S.D.)	7613	24.5 (3.4)	4779	25.1 (3.5)	4422	26.3 (9.6)
Overweight (% yes)	7613	37.6	4779	46.3	4422	56.6
Psychological distress (% yes)	7929	27.1	6029	21.7	5487	21.1
Relationship problems (% yes)	5051	30.1	5433	32.3	5439	24.6

S.D., Standard deviation.

a Number of person-observations.

b Age at the beginning of incident CHD follow-up.

Serially adjusted associations of repeat measurement of CHD risk factors and the incidence of CHD are presented in Table 2. Onset, reversed and persistent high cholesterol and blood pressure levels were associated with increased risk of CHD when compared to having normal cholesterol or blood pressure levels at both times (HRs ranging from 1.27 to 1.79 in the multivariable adjusted models). Regarding smoking, overweight, psychological distress and relationship problems, only persistent exposure was associated with CHD risk, when compared with persistent non-exposure to these risk factors (HRs ranging from 1.43 to 1.82). The highest absolute risk of CHD (incidence per 1000 person-years) was observed for participants with persistently high blood pressure (13.2), followed by reversed (12.3) and onset (11.3) of high blood pressure, and persistent smoking (10.5). The lowest absolute risk was found for participants with persistent normal BMI (5.4), persistent normal cholesterol level (5.5) and persistent normal blood pressure (5.7).

**Table 2:** Risk factors at two consecutive study waves as predictors of the incidence of CHD at follow-up.

Risk factors at two consecutive study waves	n of observations/n of CHD events	Unadjusted incidence/1000 person-years	Hazard ratio (95% CI)^a^	Hazard ratio (95% CI)^b^	Hazard ratio (95% CI)^c^
Cholesterol level					
Normal	8020/236	5.5	1.00	1.00	1.00
Onset high	2653/103	7.0	1.54 (1.22–1.95)	1.56 (1.23–1.96)	1.59 (1.26–2.00)
Reversed	2307/109	9.1	1.29 (1.03–1.62)	1.30 (1.03–1.63)	1.27 (1.01–1.60)
Persistent high	5039/265	9.8	1.74 (1.46–2.07)	1.76 (1.48–2.10)	1.79 (1.50–2.13)
Hypertension					
Normal	13,289/407	5.7	1.00	1.00	1.00
Onset	1914/113	11.3	1.68 (1.36–2.08)	1.67 (1.35–2.06)	1.64 (1.33–2.03)
Reversed	1441/93	12.3	1.87 (1.49–2.35)	1.86 (1.48–2.33)	1.76 (1.40–2.21)
Persistent	1582/109	13.2	1.81 (1.46–2.25)	1.77 (1.42–2.20)	1.72 (1.38–2.14)
Smoking					
Never smoker	9751/336	6.4	1.00	1.00	1.00
Persistent ex-smoker	6477/263	7.6	1.06 (0.90–1.24)	1.10 (0.93–1.30)	1.08 (0.92–1.28)
Smoking relapse/onset	403/18	8.7	1.09 (0.68–1.74)	1.11 (0.69–1.78)	1.09 (0.68–1.75)
Quitter	610/19	5.9	0.83 (0.52–1.32)	0.85 (0.53–1.35)	0.83 (0.52–1.32)
Persistent smoker	1898/106	10.5	1.83 (1.47–2.28)	1.84 (1.47–2.29)	1.82 (1.46–2.28)
Overweight					
No	7231/212	5.4	1.00	1.00	1.00
Onset	2018/63	5.8	1.06 (0.80–1.40)	1.06 (0.80–1.40)	1.04 (0.79–1.38)
Reversed	490/17	6.5	1.05 (0.64–1.72)	1.02 (0.62–1.67)	1.00 (0.61–1.64)
Persistent	7099/384	10.3	1.62 (1.37–1.93)	1.60 (1.35–1.90)	1.55 (1.31–1.84)
Psychological distress					
No	12,640/486	7.2	1.00	1.00	1.00
Onset	2194/70	6.0	0.99 (0.77–1.27)	0.98 (0.77–1.27)	0.93 (0.72–1.19)
Reversed	2685/97	6.7	1.10 (0.88–1.36)	1.12 (0.90–1.39)	1.08 (0.87–1.35)
Persistent	1926/103	10.0	1.78 (1.44–2.21)	1.77 (1.42–2.20)	1.63 (1.31–2.03)
Relationship problems					
No	9816/394	6.7	1.00	1.00	1.00
Onset	1981/78	6.2	1.07 (0.84–1.36)	1.01 (0.79–1.29)	0.99 (0.78–1.27)
Reversed	2458/93	6.4	0.99 (0.79–1.24)	0.95 (0.76–1.19)	0.93 (0.74–1.17)
Persistent	2367/138	9.3	1.59 (1.31–1.93)	1.46 (1.20–1.78)	1.43 (1.17–1.75)

a Model 1: adjusted for age and sex.

b Model 2: as model 1 and additionally adjusted for socioeconomic status, ethnicity, and marital status.

c Model 3: as model 2 and additionally adjusted for self-reported longstanding illness.

Comparisons between participants with reversed risk factors and those with persistent exposure to risk factors are presented in Table 3. Compared with persistent exposure, reversed high cholesterol level was associated with a significantly reduced risk (HR = 0.73). Similarly, quitting smoking (HR = 0.49), reversed psychological distress (HR = 0.68) and reversed relationship problems (HR = 0.65) were associated with a lower risk of CHD when compared to persistent exposure to the risk factor. Reversed high blood pressure (HR = 0.99) was not associated with reduced CHD risk and for reversed overweight, the association was non-significant (HR = 0.65, 95% CI 0.40–1.05). We further analyzed the mean systolic and diastolic blood pressure among the reversed versus persistently high blood pressure groups. At Tx, the mean systolic blood pressure was 141.0 mm Hg in the reversed group and 145.2 mm Hg in the persistent group (*P* for difference < 0.001); the mean diastolic blood pressure was 90.2 mm Hg (reversed group) and 91.0 mm Hg (persistent group; *P* for difference = 0.011). At Tx + 1, the mean systolic blood pressure was 126.1 mm Hg (reversed group) and 147.9 mm Hg (persistent group; *P* < 0.001). The mean diastolic blood pressure was 79.5 mm Hg (reversed group) and 90.4 mm Hg (persistent group; *P* < 0.001).

**Table 3:** Comparison between persistent (High-High) and risk factor reversal (High-Normal) at two consecutive study waves predicting the incidence of CHD at follow-up.

Risk factors at two consecutive study waves	Hazard ratio (95% CI)^a^	Hazard ratio (95% CI)^b^	Hazard ratio (95% CI)^c^
Cholesterol level			
Persistent high	1.00	1.00	1.00
Reversed	0.76 (0.61–0.95)	0.75 (0.60–0.94)	0.73 (0.58–0.91)
Hypertension			
Persistent	1.00	1.00	1.00
Reversed	1.01 (0.76–1.33)	0.99 (0.75–1.31)	0.99 (0.75–1.31)
Smoking			
Persistent smoking	1.00	1.00	1.00
Quitting	0.46 (0.28–0.77)	0.49 (0.29–0.83)	0.49 (0.29–0.82)
Overweight			
Persistent overweight	1.00	1.00	1.00
Reversed	0.64 (0.40–1.04)	0.64 (0.39–1.03)	0.65 (0.40–1.05)
Psychological distress			
Persistent distress	1.00	1.00	1.00
Reversed	0.61 (0.46–0.80)	0.63 (0.48–0.84)	0.68 (0.51–0.90)
Relationship problems			
Persistent problems	1.00	1.00	1.00
Improved	0.63 (0.48–0.82)	0.65 (0.50–0.85)	0.65 (0.50–0.85)

a Model 1: adjusted for age and sex.

b Model 2: as model 1 and additionally adjusted for socioeconomic status, ethnicity, and marital status.

c Model 3: as model 2 and additionally adjusted for longstanding illness.

Persistent exposure to both of the two biological risk factors (high cholesterol and hypertension) was associated with a 3.22-fold CHD risk when compared with persistent non-exposure to either of the two biological risk factors (Table 4). Similarly, persistent exposure to both of the two behavioral risk factors was associated with a 3.63-fold risk and persistent exposure to both of the two psychosocial factors with a 2.20-fold risk when compared with persistent non-exposure. A significant linear trend was observed to suggest a dose-response relationship. The highest absolute CHD risk was observed for persistent exposure to both of the two biological risk factors (incidence 18.1/1000 person-years) and persistent exposure to both of the two behavioral risk factors (17.7) while the lowest risk was found for persistent non-exposure to biological (4.4) and behavioral factors (4.7). Persistent exposure (12.5) and non-exposure (7.0) to psychosocial risk factors fell in-between these extremes.

**Table 4:** Association between persistent or repeated exposure to biological, behavioral and psychosocial risk factors and the incidence of CHD.

Risk factors at two consecutive study waves	n of observations/n of CHD events	Unadjusted incidence/1000 person-years	Hazard ratio (95% CI)^a^	Hazard ratio (95% CI)^b^	Hazard ratio (95% CI)^c^
Biological risk factors (high cholesterol and hypertension)					
Persistently unexposed to neither	6118/144	4.4	1.00	1.00	1.00
Onset of either	4273/197	8.5	1.89 (1.53–2.35)	1.90 (1.53–2.35)	1.89 (1.53–2.35)
Persistently exposed to both	554/52	18.1	3.29 (2.37–4.56)	3.27 (2.36–4.55)	3.22 (2.32–4.48)
*P* for trend			<0.001	<0.001	<0.001
Behavioral risk factors (smoking^d^ and overweight)					
Persistently unexposed to neither	3827/99	4.7	1.00	1.00	1.00
Onset of either	2390/81	6.3	1.21 (0.90–1.62)	1.23 (0.92–1.66)	1.20 (0.89–1.62)
Persistently exposed to both	641/59	17.7	3.55 (2.57–4.91)	3.74 (2.68–5.21)	3.63 (2.60–5.06)
*P* for trend			<0.001	<0.001	<0.001
Psychosocial risk factors (psychological distress and relationship problems)					
Persistently unexposed to neither	6837/249	7.0	1.00	1.00	1.00
Onset of either	3777/133	6.6	1.18 (0.96–1.46)	1.13 (0.91–1.41)	1.10 (0.88–1.36)
Persistently exposed to both	441/29	12.5	2.42 (1.64–3.56)	2.33 (1.58–3.43)	2.20 (1.49–3.26)
*P* for trend			<0.001	0.002	0.007

a Model 1: adjusted for age and sex.

b Model 2: as model 1 and additionally adjusted for socioeconomic status, ethnicity, and marital status.

c Model 3: as model 2 and additionally adjusted for longstanding illness.

d Unexposed includes never-smokers only.

In a sensitivity analysis, we further adjusted the models for study cycle but this had little effect on the estimates. The association for reversed cholesterol level (as presented in Table 3; not shown in tables) slightly weakened (HR = 0.82, 95% CI 0.64–1.04) and the association for reversed overweight slightly strengthened (HR = 0.62, 95% CI 0.39–1.01).

Supplemental Table 2, Supplemental Table 3, Supplemental Table 4 show the results from other sensitivity analyses carried out among participants aged >60 years in the beginning of follow-up. The results were rather similar to those among the total population although the associations with biological risk factors seemed somewhat weaker.

## 4 Discussion

In this large cohort study of middle-aged British men and women, we used observational data to create a non-randomized pseudo-trial to assess temporality between change in CHD risk factors and subsequent incidence of CHD. To our knowledge, comparison of favorable and unfavorable changes in biological, behavioral and psychosocial risk factors in a large CHD-free population has not previously been done. The onset of high cholesterol and high blood pressure but not behavioral or psychosocial factors, increased the risk of CHD and persistent or repeated exposure to all measured risk factors was associated with an increased risk, when compared with persistent or repeated non-exposure to the risk factor. However, reduced cholesterol level, psychological distress, relationship problems, and quitting smoking were associated with a lower risk of CHD when compared with persistent or repeated exposure to those risk factors, whereas reversed hypertension did not decrease the risk.

Risk factors can be divided into proximal and distal factors by how close they are to being the actual cause of the disease [17]. Interestingly, only the onset of high cholesterol and hypertension (i.e., biological risk factors) but not the onset of behavioral or psychosocial factors was associated with the incidence of CHD. As biological factors are ‘proximal’, the research evidence of their contribution to CHD might be more consistent [4,17]. More distal behavioral and psychosocial factors might require longer latency and therefore have a weaker effect on CHD in a 5-year follow-up, since they have been suggested to operate through changes in proximal factors [17,18]. For smoking, the average success rate of quitting smoking is 10% [19], thus quitting and relapse might take turns between survey waves and lead to some misclassification of smoking exposure.

We found that participants whose exposure to high cholesterol, psychological distress, or relationship problems was reversed and those who quit smoking had an approximately 0.5 to 0.7-fold lower risk of CHD when compared with persistent exposure to the risk factor in question. For overweight, the estimate did not reach statistical significance although the magnitude (hazard ratio 0.65) was similar to that of other risk factors. These findings suggest that reducing these risk factors to recommended levels might be beneficial in preventing the onset of CHD, in agreement with the strong evidence base showing beneficial effect of reducing cholesterol and blood pressure [[4], [5], [6], [7]], quitting smoking [4,5], and reducing body weight [4,20]. One observational study examined time since quitting and found a hazard ratio of 0.5 for myocardial infarction with 5 to 9 years since cessation [21]. Also in line with our findings, one observational study found no significant association between changes in BMI and cardiovascular mortality during 11 years of follow-up [22].

Previous research has shown that mental health symptoms, such as depression, high stress, and perceived problems in social relationships might increase the risk of CHD [8,9,18,23,24]. We add to the existing evidence by demonstrating that participants whose psychosocial exposures were resolved had a lower risk of CHD when compared with their counterparts with persistent or repeated problems. Both direct mechanisms, i.e., altered physiological responses and inflammation, and indirect mechanisms, i.e., health behaviors, have been suggested to explain the link between these psychosocial factors and CHD [8,25]. Previous studies using Whitehall II data suggest that long-term but not short-term psychological distress is associated with subclinical coronary artery calcification [26], and that negative aspects in social relationships increase BMI and waist circumference [16], whereas recovery from psychological distress might reduce interleukin-6 levels [27].

Surprisingly, hypertension appeared to be related to a heightened CHD risk even if it was alleviated. Meta-analyses of clinical trials have shown that antihypertensive treatment significantly reduces the risk of heart disease in various study populations [28], but in observational data reductions in blood pressure could also result from adverse changes in health, such as disease-related unintentional weight loss. One meta-analysis assessed blood pressure lowering trials and found only 5 studies with CHD-free populations. The effect estimate for CHD incidence was statistically non-significant [28]. In another meta-analysis focusing on an intermediate-risk population without prevalent cardiovascular disease, a clear beneficial effect was shown with a combination of antihypertensive and lipid-lowering therapy but not with antihypertensive therapy alone [7]. Furthermore, there is evidence of ‘residual cardiovascular risk’ in individuals on blood pressure –lowering treatment, i.e., they have been shown to have a higher risk of experiencing a cardiovascular event than those who are untreated but have similar (normal) blood pressure levels [29]. This increased risk has been shown to be attributable in part to greater subclinical disease burden among treated individuals [29,30].

Our comparison of absolute risk of CHD events between single risk factors showed the highest absolute risk to be associated with the onset (11.3/1000 person-years), reversed (12.3) and persistent (13.2) hypertension. The risk associated with persistent exposure to other risk factors varied between 9.3 and 10.5. Thus, our findings particularly support the importance of early prevention of hypertension as an important target in the primary prevention of CHD. However, among the groups of biological, behavioral and psychosocial risks, the highest absolute risk was found for those with persistent exposure to both hypertension and high cholesterol (18.1) as well as those with persistent exposure to both smoking and overweight (17.7) which highlights the importance of addressing multiple risks [4].

### 4.1 Strengths and limitations

The specific strengths of this study are its large number of observations over several study waves and reliable clinical assessments of CHD, blood pressure, cholesterol, and BMI. The pseudo-trial design allowed us to reduce bias related to unmeasured time-invariant confounding factors. However, although the non-randomized pseudo trial study design has many advantages, we were not able to control for exposures changing during the follow-up (time-varying confounders), such as uptake of new pharmacological treatments resulting in better blood pressure control or adverse changes in blood pressure due to increasing morbidity, non-adherence to treatments, or lifestyle changes. Unmeasured adverse conditions at follow-up might explain why a decline of blood pressure from high levels was associated with an increased risk of CHD. An ideal study for future studies would include close monitoring of time-varying exposures at follow-up.

One of the limitations is that although the study had a high response rate in the successive data collection waves, loss to follow-up accumulated, as is the case in most long-term cohort studies. Our assessments of smoking, psychological distress and close relationships were based on self-report which may be affected, for example by personal response styles. However, when we examined change in these exposures, response style was an unlikely explanation for the associations observed in our study. As the number of women was relatively low, we were not able to examine whether associations were different between men and women. As in all observational studies, the possibility of residual confounding, referring to unmeasured factors, such as specific biomarkers [31,32], cannot be ruled out. Lastly, participants of the Whitehall II study are from an occupational cohort and mainly white, which somewhat limits generalizability of our findings.

## 5 Conclusions

The persistent high levels of biological, behavioral and psychological risk factors were associated with increased 5-year risk of CHD in participants initially free of CHD. The greatest risk was found among participants with long-term exposure to multiple risk factors. Among those who already had high risk factor levels, improvement in four out of the six risk factors examined (total cholesterol, psychological distress, social relationships, and quitting smoking) reduced the risk of CHD. The observed favorable changes in risk factors followed by reduced CHD suggest relatively rapid benefits from the reduction of these risk factors.

The following are the supplementary data related to this article.

Supplemental Fig. 1 Setting of three nested cohorts (pseudo-trials) used in analyses of the incidence of coronary heart disease in the Whitehall II Study (n = 20,357 person-observations). Supplemental Fig. 1

Supplemental Fig. 2 Flow chart of the sample selection procedure for the main analysis. Supplemental Fig. 2

Supplemental Table 1 Descriptive statistics of the participants at the Whitehall II study entry and at the beginning of follow-up across the 3 nested cohorts. Supplemental Table 1

Supplemental Table 2 Association between biological, behavioral and psychosocial risk factors at two consecutive study waves and the incidence of CHD among participants aged >60 years. Supplemental Table 2

Supplemental Table 3 Comparison between persistent (High-High) and risk factor reversal (High-Normal) at two consecutive study waves predicting the incidence of CHD at follow-up among participants aged >60 years. Supplemental Table 3

Supplemental Table 4 Association between long-term or repeated exposure to biological, behavioral and psychosocial risk factors and the incidence of CHD among participants aged >60 years. Supplemental Table 4

## Grant support

This work was supported by the Academy of Finland (258598, 292824, 265977, 267727, 311492), the UK Medical Research Council (K013351), and NordForsk (75021); the Nordic Research Program on Health and Welfare. The UK Medical Research Council (K013351, MRR024227), British Heart Foundation, and the US National Institutes of Health (R01HL36310, R01AG013196) have supported collection of data in the Whitehall II Study.

## Conflict of interest

The authors report no relationships that could be construed as a conflict of interest.
