# Supplementary material for: Unfavorable and favorable changes in modifiable risk factors and incidence of coronary heart disease: The Whitehall II cohort study
Source: Int J Cardiol. 2018 Oct 15;269:7–12. doi: 10.1016/j.ijcard.2018.07.005 (PMC6152587; doi:10.1016/j.ijcard.2018.07.005)
Supplement: Supplemental Fig. 1 — Setting of three nested cohorts (pseudo-trials) used in analyses of the incidence of coronary heart disease in the Whitehall II Study (n = 20,357 person-observations). [file mmc1.pdf]

Supplemental Figure 1. Setting of three nested cohorts (pseudo-trials) used in analyses of the incidence of coronary heart disease in the Whitehall II study (n=20,357 person-observations)

Nested cohort	Person-observations	Person-years	Events	Rate[a]
1	8012	45,556	291	6.4
2	6336	33,559	208	6.2
3	6009	28,792	300	10.4

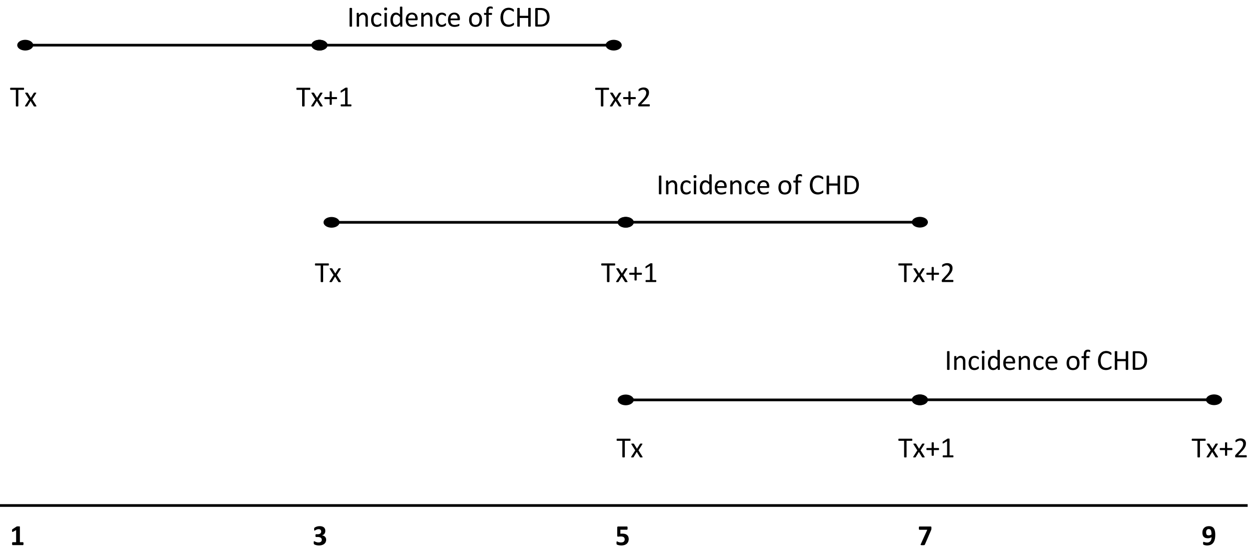

Whitehall II study wave

Tx: First assessment of exposure to risk factors among participants free from CHD.
Tx+1: Second assessment of exposure to risk factors among participants free from CHD.
Time between Tx+1 and Tx+2: Follow-up for the incidence of CHD.
[a]Unadjusted incidence of CHD per 1000 person-years.
