# Supplementary figures and images for: Unfavorable and favorable changes in modifiable risk factors and incidence of coronary heart disease: The Whitehall II cohort study
Source: Int J Cardiol. 2018 Oct 15;269:7–12. doi: 10.1016/j.ijcard.2018.07.005 (PMC6152587; doi:10.1016/j.ijcard.2018.07.005)

**Supplemental Figure 2.** Flow chart of the sample selection procedure for the main analysis

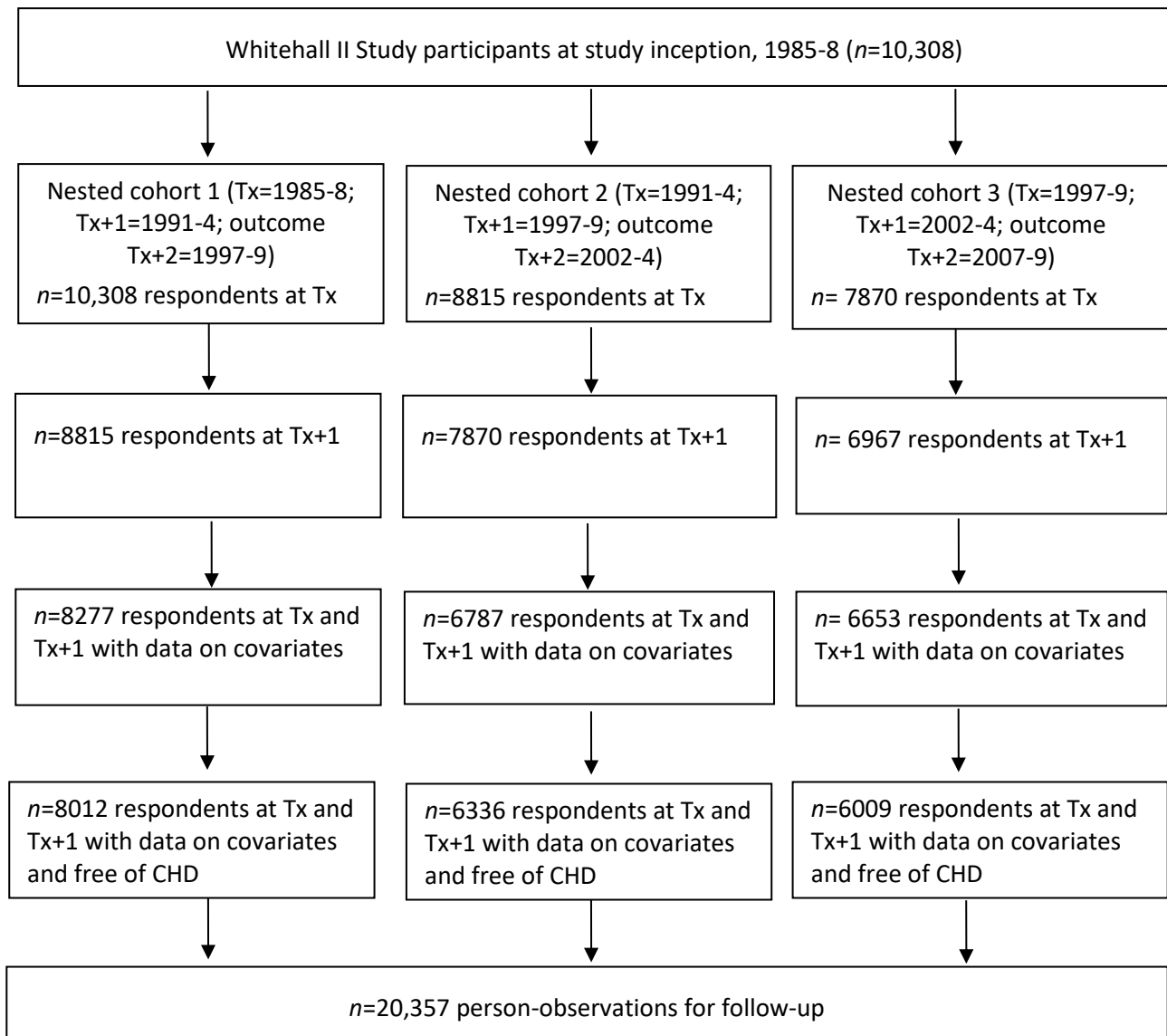

Supplement: Supplemental Fig. 2 — Flow chart of the sample selection procedure for the main analysis. [file mmc2.pdf]
