# Supplementary material for: Unfavorable and favorable changes in modifiable risk factors and incidence of coronary heart disease: The Whitehall II cohort study
Source: Int J Cardiol. 2018 Oct 15;269:7–12. doi: 10.1016/j.ijcard.2018.07.005 (PMC6152587; doi:10.1016/j.ijcard.2018.07.005)
Supplement: Supplemental Table 1 — Descriptive statistics of the participants at the Whitehall II study entry and at the beginning of follow-up across the 3 nested cohorts. [file mmc3.docx]

**Supplemental Table 1.** Descriptive statistics of the participants at the Whitehall II study entry and at the beginning of follow-up across the 3 nested cohorts

|  | All study participants without CHD at study entry (1985-8)  n= 10,187 | The nested cohort I at study entry (1985-8)  n=8012 | The nested cohort II at study entry (1985-8)  n=6336 | The nested cohort III at study entry (1985-8)  n=6009 |
| --- | --- | --- | --- | --- |
| Characteristics | n (%) / mean (SD) | n (%) / mean (SD) | n (%) / mean (SD) | n (%) / mean (SD) |
| Age (mean, SD) | 44.4 (6.0) | 44.2 (6.0) | 44.1 (6.0) | 43.9 (5.9) |
| Sex (n, %): men | 6800 (66.8) | 5525 (69.0) | 4476 (70.6) | 4208 (70.0) |
| women | 3387 (33.3) | 2487 (31.0) | 1860 (29.4) | 1801 (30.0) |
| Socioeconomic status (n, %): high | 2994 (29.4) | 2515 (31.4) | 2152 (34.0) | 2025 (33.7) |
| intermediate | 4894 (48.0) | 3972 (49.6) | 3166 (50.0) | 3035 (50.5) |
| low | 2299 (22.6) | 1525 (19.0) | 1018 (16.1) | 949 (15.8) |
| Race/ ethnicity (n, %): white | 9063 (89.8) | 7286 (90.9) | 5837 (92.1) | 5564 (92.6) |
| non-white | 1033 (10.2) | 726 (9.1) | 499 (7.9) | 445 (7.4) |
| Marital status (n, %): married /cohabiting | 7520 (74.1) | 6019 (75.4) | 4925 (78.0) | 4568 (76.2) |
| non-married /-cohabiting | 2630 (25.9) | 1965 (24.6) | 1388 (22.0) | 1426 (23.8) |
| Self-reported longstanding illness^a^ (n, %): no | 6503 (67.7) | 5301 (68.5) | 4234 (69.1) | 4022 (69.1) |
| yes | 3101 (32.3) | 2440 (31.5) | 1897 (30.9) | 1795 (30.9) |

^a^The number of responses to longstanding illness at study entry was lower because the question was introduced after the beginning of data collection. Missing responses (26%) were completed by phase 2 responses when possible.
