# Supplementary material for: Unfavorable and favorable changes in modifiable risk factors and incidence of coronary heart disease: The Whitehall II cohort study
Source: Int J Cardiol. 2018 Oct 15;269:7–12. doi: 10.1016/j.ijcard.2018.07.005 (PMC6152587; doi:10.1016/j.ijcard.2018.07.005)
Supplement: Supplemental Table 2 — Association between biological, behavioral and psychosocial risk factors at two consecutive study waves and the incidence of CHD among participants aged >60 years. [file mmc4.docx]

**Supplemental Table 2**. Association between biological, behavioral and psychosocial risk factors at two consecutive study waves and the incidence of CHD among participants aged >60 years

| Risk factors at two consecutive study waves | n of observations / n of CHD events | Unadjusted incidence / 1000 person-years | Hazard ratio (95% CI)^a^ |
| --- | --- | --- | --- |
| Cholesterol level |  |  |  |
| Normal | 1746 / 105 | 12.2 | 1.00 |
| Onset high | 299 / 23 | 15.7 | 1.45 (0.93-2.27) |
| Reversed | 886 / 65 | 14.5 | 1.25 (0.91-1.70) |
| Persistent high | 1116 / 82 | 14.7 | 1.46 (1.09-1.96) |
| Hypertension |  |  |  |
| Normal | 2580 / 147 | 11.4 | 1.00 |
| Onset | 588 / 48 | 16.6 | 1.42 (1.02-1.98) |
| Reversed | 382 / 34 | 18.3 | 1.42 (0.97-2.08) |
| Persistent | 562 / 50 | 18.2 | 1.41 (1.02-1.95) |
| Smoking |  |  |  |
| Never smoker | 2148 / 128 | 12.0 | 1.00 |
| Persistent ex-smoker | 1737 / 104 | 12.1 | 0.93 (0.71-1.21) |
| Smoking relapse/onset | 125 / 9 | 14.6 | 1.00 (0.51-1.95) |
| Quitter | 161 / 8 | 10.0 | 0.77 (0.38-1.58) |
| Persistent smoker | 319 / 28 | 18.2 | 1.67 (1.10-2.54) |
| Overweight |  |  |  |
| No | 1261 / 74 | 11.7 | 1.00 |
| Onset | 339 / 23 | 13.5 | 1.10 (0.69-1.76) |
| Reversed | 124 / 5 | 8.1 | 0.71 (0.29-1.71) |
| Persistent | 1860 / 148 | 16.2 | 1.33 (1.00-1.76) |
| Psychological distress |  |  |  |
| No | 3446 / 207 | 12.1 | 1.00 |
| Onset | 400 / 18 | 9.2 | 0.77 (0.48-1.26) |
| Reversed | 457 / 33 | 14.6 | 1.30 (0.91-1.88) |
| Persistent | 277 / 28 | 20.7 | 1.75 (1.17-2.63) |
| Relationship problems |  |  |  |
| No | 2864 / 163 | 11.5 | 1.00 |
| Onset | 389 / 24 | 12.1 | 1.06 (0.69-1.64) |
| Reversed | 619 / 40 | 12.9 | 1.07 (0.76-1.51) |
| Persistent | 486 / 48 | 20.2 | 1.63 (1.17-2.28) |

^a^Adjusted for age, sex, socioeconomic status, ethnicity, marital status, and longstanding illness.
