# Supplementary material for: Unfavorable and favorable changes in modifiable risk factors and incidence of coronary heart disease: The Whitehall II cohort study
Source: Int J Cardiol. 2018 Oct 15;269:7–12. doi: 10.1016/j.ijcard.2018.07.005 (PMC6152587; doi:10.1016/j.ijcard.2018.07.005)
Supplement: Supplemental Table 3 — Comparison between persistent (High-High) and risk factor reversal (High-Normal) at two consecutive study waves predicting the incidence of CHD at follow-up among participants aged >60 years. [file mmc5.docx]

**Supplemental Table 3.** Comparison between persistent (High-High) and risk factor reversal (High-Normal) at two consecutive study waves predicting the incidence of CHD at follow-up among participants aged >60 years

| Risk factors at two consecutive study waves | Hazard ratio (95% CI)^a^ |
| --- | --- |
| Cholesterol level |  |
| Persistent high | 1.00 |
| Reversed | 0.88 (0.63-1.22) |
| Hypertension |  |
| Persistent | 1.00 |
| Reversed | 0.99 (0.64-1.53) |
| Smoking |  |
| Persistent smoking | 1.00 |
| Quitting | 0.48 (0.20-1.14) |
| Overweight |  |
| Persistent overweight | 1.00 |
| Reversed | 0.53 (0.22-1.25) |
| Psychological distress |  |
| Persistent distress | 1.00 |
| Reversed | 0.81 (0.48-1.37) |
| Relationship problems |  |
| Persistent problems | 1.00 |
| Improved | 0.65 (0.42-1.01) |

^a^Adjusted for age, sex, socioeconomic status, ethnicity, marital status, and longstanding illness.
