# Supplementary material for: Unfavorable and favorable changes in modifiable risk factors and incidence of coronary heart disease: The Whitehall II cohort study
Source: Int J Cardiol. 2018 Oct 15;269:7–12. doi: 10.1016/j.ijcard.2018.07.005 (PMC6152587; doi:10.1016/j.ijcard.2018.07.005)
Supplement: Supplemental Table 4 — Association between long-term or repeated exposure to biological, behavioral and psychosocial risk factors and the incidence of CHD among participants aged >60 years. [file mmc6.docx]

**Supplemental Table 4**. Association between long-term or repeated exposure to biological, behavioral and psychosocial risk factors and the incidence of CHD among participants aged >60 years

| Risk factors at two consecutive study waves | n of observations / n of CHD events | Unadjusted incidence / 1000 person-years | Hazard ratio (95% CI)^a^ |
| --- | --- | --- | --- |
| Biological risk factors (high cholesterol and hypertension) |  |  |  |
| Persistently unexposed to neither | 1124 / 59 | 10.6 | 1.00 |
| Onset of either | 837 / 63 | 15.3 | 1.53 (1.07-2.18) |
| Persistently exposed to both | 174 / 16 | 18.9 | 1.82 (1.04-3.21) |
| p for trend |  |  | 0.006 |
| Behavioral risk factors (smoking^b^ and overweight) |  |  |  |
| Persistently unexposed to neither | 616 / 29 | 9.3 | 1.00 |
| Onset of either | 457 / 32 | 14.0 | 1.43 (0.86-2.38) |
| Persistently exposed to both | 126 / 19 | 32.2 | 4.07 (2.20-7.52) |
| p for trend |  |  | <0.001 |
| Psychosocial risk factors (psychological distress and relationship problems) |  |  |  |
| Persistently unexposed to neither | 2258 / 120 | 10.8 | 1.00 |
| Onset of either | 727 / 38 | 10.6 | 1.08 (0.74-1.56) |
| Persistently exposed to both | 74 / 8 | 22.1 | 2.27 (1.08-4.76) |
| p for trend |  |  | 0.15 |

^a^Adjusted for age, sex, socioeconomic status, ethnicity, marital status, and longstanding illness.

^b^Unexposed includes never-smokers only.
